# Helium Ion Therapy for Advanced Juvenile Nasopharyngeal Angiofibroma

**DOI:** 10.3390/cancers16111993

**Published:** 2024-05-24

**Authors:** Line Hoeltgen, Eva Meixner, Philipp Hoegen-Saßmannshausen, Ji-Young Kim, Maximilian Deng, Katharina Seidensaal, Thomas Held, Klaus Herfarth, Thomas Haberer, Jürgen Debus, Andrea Mairani, Semi Harrabi, Thomas Tessonnier

**Affiliations:** 1 Department of Radiation Oncology, Heidelberg University Hospital, 69120 Heidelberg, Germany; line.hoeltgen@med.uni-heidelberg.de (L.H.);; 2 Heidelberg Ion-Beam Therapy Center (HIT), Heidelberg University Hospital, 69120 Heidelberg, Germany; 3 National Center for Tumor Diseases (NCT), 69120 Heidelberg, Germany; 4 Clinical Cooperation Unit Radiation Oncology, German Cancer Research Center (DKFZ), 69120 Heidelberg, Germany; 5 Heidelberg Institute of Radiation Oncology (HIRO), 69120 Heidelberg, Germany; 6 Partner Site, German Cancer Consortium (DKTK), 69120 Heidelberg, Germany; 7 Centro Nazionale di Adroterapia Oncologica (CNAO), Medical Physics Department, 27100 Pavia, Italy

**Keywords:** juvenile nasopharyngeal angiofibroma (JNA), pediatrics, proton therapy, helium ion therapy, NTCP, secondary cancers

## Abstract

**Simple Summary:**

Juvenile nasopharyngeal angiofibroma (JNA) presents a therapeutic challenge in its advanced stages due to the close proximity to critical structures at the skull base. While radiotherapy is an effective treatment alternative, radiation-induced complications due to dose deposition in healthy tissue remain a major concern. Particle radiotherapy offers a high degree of conformality, and notably, helium ions present a promising treatment option due to their favorable biophysical characteristics between protons and carbon ions, supporting their reconsideration for clinical application. We retrospectively designed helium ion therapy (HRT) plans for all patients previously treated with proton therapy (PRT) at the Heidelberg Ion-Beam Therapy Center. Our findings indicate potential dosimetric advantages of HRT over PRT for advanced JNA, suggesting enhanced target coverage along with reduced dose deposition in healthy tissue, thereby potentially mitigating acute and late-term complications, including secondary neoplasms. This is particularly important given the benign nature of JNA and the patients’ young age.

**Abstract:**

Helium ion therapy (HRT) is a promising modality for the treatment of pediatric tumors and those located close to critical structures due to the favorable biophysical properties of helium ions. This in silico study aimed to explore the potential benefits of HRT in advanced juvenile nasopharyngeal angiofibroma (JNA) compared to proton therapy (PRT). We assessed 11 consecutive patients previously treated with PRT for JNA in a definitive or postoperative setting with a relative biological effectiveness (RBE) weighted dose of 45 Gy (RBE) in 25 fractions at the Heidelberg Ion-Beam Therapy Center. HRT plans were designed retrospectively for dosimetric comparisons and risk assessments of radiation-induced complications. HRT led to enhanced target coverage in all patients, along with sparing of critical organs at risk, including a reduction in the brain integral dose by approximately 27%. In terms of estimated risks of radiation-induced complications, HRT led to a reduction in ocular toxicity, cataract development, xerostomia, tinnitus, alopecia and delayed recall. Similarly, HRT led to reduced estimated risks of radiation-induced secondary neoplasms, with a mean excess absolute risk reduction of approximately 30% for secondary CNS malignancies. HRT is a promising modality for advanced JNA, with the potential for enhanced sparing of healthy tissue and thus reduced radiation-induced acute and long-term complications.

## 1. Introduction

Juvenile nasopharyngeal angiofibroma (JNA) is a rare benign but locally aggressive condition, which affects almost exclusively young men [1]. Advanced stages of JNA with intracranial involvement present a therapeutic challenge due to their close proximity to critical structures at the skull base, posing difficulties in achieving complete surgical removal [2]. In incompletely resected JNA or tumors that are deemed irresectable without major morbidity, radiotherapy is known to be an effective treatment alternative [3,4]. However, radiation-induced long-term complications remain a major concern due to the generally young age of patients affected by JNA as well as the benign nature of the tumor [1].

Compared to conventional photon radiotherapy, particle radiotherapy holds potential in reducing such long-term sequelae by preserving surrounding healthy tissue more effectively, owing to the favorable biophysical characteristics of particles [5]. In current clinical particle therapy for pediatric tumors, predominantly protons are used, and to a lesser extent, carbon ions for certain indications [6,7].

The first clinical report on proton radiotherapy (PRT) for advanced JNA has shown excellent local control and patient outcomes, with potentially reduced radiation-induced long-term complications compared to conventional radiotherapy [8]. The use of carbon ions in younger patients remains disputed. Although offering a steeper lateral dose gradient compared to lighter particles such as protons, carbon ions present a higher linear energy transfer and fragmentation tail composed of secondary lighter particles and neutrons, leading to unwanted doses after the Bragg peak [9].

Displaying favorable intermediate biophysical characteristics between protons and carbon ions, helium ions are gaining increasing interest, notably in pediatric cases. They exhibit a reduced fragmentation tail and less uncertainty in RBE than carbon ions, along with reduced lateral scattering and a higher RBE compared to protons [9,10]. Despite their successful utilization in pioneering clinical studies at LBNL several decades ago, treating over 2000 patients with various tumor types, helium ions have not been integrated into clinical routine, where protons and to a minor extent carbon ions have taken precedence [11].

Yet, helium ions are currently being considered for reintroduction into clinical practice, recognizing their potential benefits over protons and carbon ions [11]. Indeed, they hold promise in potentially enhancing treatment effectiveness and sparing healthy tissue across diverse tumor sites [10,12,13,14], potentially further reducing radiation-induced long-term complications. This holds particular significance in the treatment of advanced JNA which are typically localized close to critical structures essential for patients’ quality of life, not least considering the generally young age of the affected patients and their considerable life expectancy.

This in silico study aims to evaluate the potential dosimetric advantages of helium ion therapy (HRT) compared to PRT for advanced JNA. In a second step, it aims to conduct risk assessments for long-term complications through normal tissue complication probability (NTCP) comparisons.

## 2. Materials and Methods

### 2.1. Patient Selection

We assessed all 11 consecutive patients who underwent PRT for JNA in a definitive or postoperative setting at the Heidelberg Ion-Beam Therapy Center between 2012 and 2023 for this in silico study. Relevant patient characteristics are depicted in Table 1. The retrospective study was conducted in accordance with the Declaration of Helsinki and approval for the analysis was granted by the Ethics Review Board of Heidelberg University (protocol code S-377).

### 2.2. Tumor and Organs at Risk Delineation

Delineation of the clinical target volume (CTV) and organs at risk (OAR) was performed according to the authors’ institutional guidelines, as previously reported [8]. Additional cerebral substructures were contoured for further evaluation of potential radiation-induced implications on neurocognitive functions according to previously published guidelines and landmarks [15,16]. The laterality of paired organs was defined according to the principal tumor area, irrespective of bilateral tumor growth.

### 2.3. Clinical Proton Treatment Planning

For patients treated prior to 2020, treatment planning for raster-scanned PRT was conducted using the treatment planning system (TPS) SyngoTPS (Siemens, Erlangen, Germany), while patients treated from 2020 onwards underwent planning using the RayStation TPS (RaySearch Laboratories AB, Stockholm, Sweden). The TPS considered a constant relative biological effectiveness value (RBE) of 1.1 for RBE-weighted dose calculation, with RBE-weighted dose expressed in Gray (Gy (RBE)) [17]. In general, 45 Gy (RBE) in fractions of 1.8 Gy (RBE) were prescribed to the CTV median dose (D_50%_). For patients receiving treatment in an additive setting, 36 Gy (RBE) was delivered to the initial tumor extension adapted to anatomic barriers, with a sequential boost of 9 Gy (RBE) to macroscopic residues. Tolerance doses for OAR were based on the quantitative analyses of normal tissue effects in the Clinic (QUANTEC) report [18]. Clinical proton treatment planning was performed based on the planning target volume (PTV), defined as a CTV with an isotropic safety margin of 3 mm. The treatment plans were designed using two to four treatment beams, either for delivery at the isocentric gantry or the fixed beamline, depending on the positioning between OAR and target volumes. All proton plans were delivered for patient treatment as reported in Hoeltgen et al. [8]. Treatment characteristics are summarized in Table 1.

### 2.4. Retrospective Helium Ion Treatment Planning

Retrospective planning of the helium plans was based on the initial planning computed tomography (CT) datasets, with targets and OAR contours from the clinically approved and delivered proton plans. The helium plans were designed using the RayStation TPS, optimized for each patient and reviewed by an experienced senior radiation oncologist. The HRT plans used the same fractionation scheme described for PRT, with 45 Gy (RBE) in 25 fractions. In contrast to protons, the helium ions RBE-weighted dose calculation employed a variable RBE model, the modified microdosimetric kinetic model (mMKM) [19], with an α/β ratio of 2 Gy (β of 0.025 Gy^−2^), as currently assigned in clinical practice [14].

The same planning strategies as used for the proton plans were used for helium planning (e.g., beam angulations, OAR dose objectives, treatment room). Planning aimed at reaching at least the same clinical goals for OAR as in the initial proton plans, but with better or similar target coverage.

### 2.5. Dosimetric Evaluation of Treatment Plans

The dose–volume histograms (DVH) of the different investigated volumes were extracted from the TPS for analysis. Evaluation of the target volume was performed using the following dosimetric parameters: D_2%_, D_5%_, D_95%_, and D_98%_ (minimum dose received in 2%, 5%, 95%, or 98% of the CTV, respectively), D_mean_ (mean CTV dose), V_95%_, V_105%_, V_107%_ (volume receiving at least 95%, 105% or 107% of the prescribed dose, respectively), conformity index (CI) and homogeneity index (HI). The HI and CI were calculated as follows:

$$HI=100\;\cdot \;\frac{D_{5\%}-D_{95\%}}{D_{p}}$$

$$CI=\frac{V_{95\%}(CTV{)}^{2}}{CTV\cdot V_{95\%}(Body)}$$

with D_p_ being the prescribed dose. V_95%_(CTV) and V_95%_(Body) correspond to the volume of the CTV and the body receiving at least 95% of the prescribed dose, respectively.

For the evaluation of OAR, various dosimetric parameters were considered, depending on the OAR, including D_mean_, D_x_ (minimum dose received in a portion x of the OAR volume), V_x_ (volume of the OAR receiving at least x Gy (RBE)) or the integral dose (ID) [20]. The ID indicates the total energy received by an OAR and was calculated as follows:

$$ID=D_{mean}\cdot V$$

with V being the volume of the respective OAR.

### 2.6. Assessment of Normal Tissue Complication Probabilities

Potential impacts on the patients’ quality of life following radiotherapy were assessed through the estimation of NTCP using different published models, as detailed in Table 2. Computation of NTCP for various OAR were performed with respect to several endpoints, including neurocognition (change in estimated intellectual quotient (IQ) [21,22] or delayed recall on the Wechsler Memory Scale-III Word List [23]), neuroendocrine dysfunctions (e.g., adrenocorticotropic or growth hormone deficiency), central hypothyroidism [24,25], hearing loss or tinnitus [25], ocular toxicity [26], alopecia or erythema [27] or xerostomia [28].

The relative risk of the development of a radiation-induced CNS malignancy following PRT compared to HRT was estimated using the concept of the risk ratio (RR). The RR represents the ratio of the excess absolute risk for radiation-induced secondary CNS malignancies between two irradiation modalities [32,33], and can be simplified as the ratio of the organ equivalent dose (OED). The RR is calculated as follows:

$$RR=\frac{{OED}_{proton}}{{OED}_{helium}}$$

$$OED=\frac{1}{V}\sum_{i}V_{i}\frac{e^{-{\alpha^{\prime }}_{i}D_{i}}}{{\alpha^{\prime }}_{i}R}\left(1-2R+R^{2}e^{{\alpha^{\prime }}_{i}D_{i}}-{\left(1-R\right)}^{2}e^{-\frac{{\alpha^{\prime }}_{i}R}{1-R}D_{i}}\right)$$

with ${\alpha^{\prime }}_{i}=\alpha +\beta \frac{D_{i}}{D_{p}}d_{T}$.

α and β correspond to the parameter of the linear quadratic model, in this case $\frac{\alpha }{\beta }=3$ Gy and α=0.018 Gy^−1^. R represents the repair/repopulation parameter, and R = 0.93. $D_{p}$ is the prescribed dose to the CTV and $d_{T}$ is the prescribed dose per fraction. $D_{i}$ and $V_{i}$ are the respective dose and volume in each bin i of the DVH of the brain. V is the total volume of the brain.

### 2.7. Statistical Analysis

Statistical analysis was conducted using MATLAB (R2009b, The MathWorks Inc., Natick, MA, USA). A two-sided Wilcoxon signed-rank test was applied for analysis and a *p* value < 0.05 was considered statistically significant.

## 3. Results

All treatment plans with helium ions met the initial objective of the clinical proton plans regarding target coverage and OAR constraints.

Along with further improved target coverage for all patients, HRT led to a lower HI, correlating with the reduced maximum dose within the target. In a subanalysis of the treatment plans without a sequential boost, a trend towards a higher CI in the HRT plans (0.73) compared to the PRT plans (0.70) was noted, while the CI did not differ between the two modalities in the whole patient cohort. Dosimetric parameters regarding the CTV are summarized in Table 3 and Supplementary Table S1.

For all OAR, the majority of considered dosimetric parameters presented a dose reduction in helium plans compared to proton plans.

Regarding the brain and its substructures, the mean dose was reduced by approximatively 1 Gy (RBE) for the infra- and supratentorial brain in HRT plans, with a mean relative difference of 26–30% in integral dose. The volume metrics for these structures (V_10_, V_15_, V_20_) were decreased by 25–35% in HRT plans, with absolute differences reaching up to approximately 4.5 Gy, as observed in the case of V_10_ for the brain (11% in PRT and 7% in HRT). The ipsilateral hippocampi were better spared in HRT plans, with a mean reduction in mean dose of approximatively 4 Gy (RBE) and a mean reduction in maximum dose of 8 Gy (RBE). Reduction of dose distribution to bilateral hippocampi (D_0.03cm^3^_, D_40%_, D_mean_) ranged from 30% to 60% in HRT plans compared to PRT plans.

For paired OAR outside the brain, helium plans allowed for an absolute mean dose reduction of 2–3 Gy (RBE), translating into an average relative reduction in mean dose of 31% for ipsilateral organs and 46% for contralateral organs. Skin irradiated volumes were reduced with helium ions, showing a mean reduction of approximately 31% and 52% in V_10_ and V_20_, respectively.

**Figure 1:**
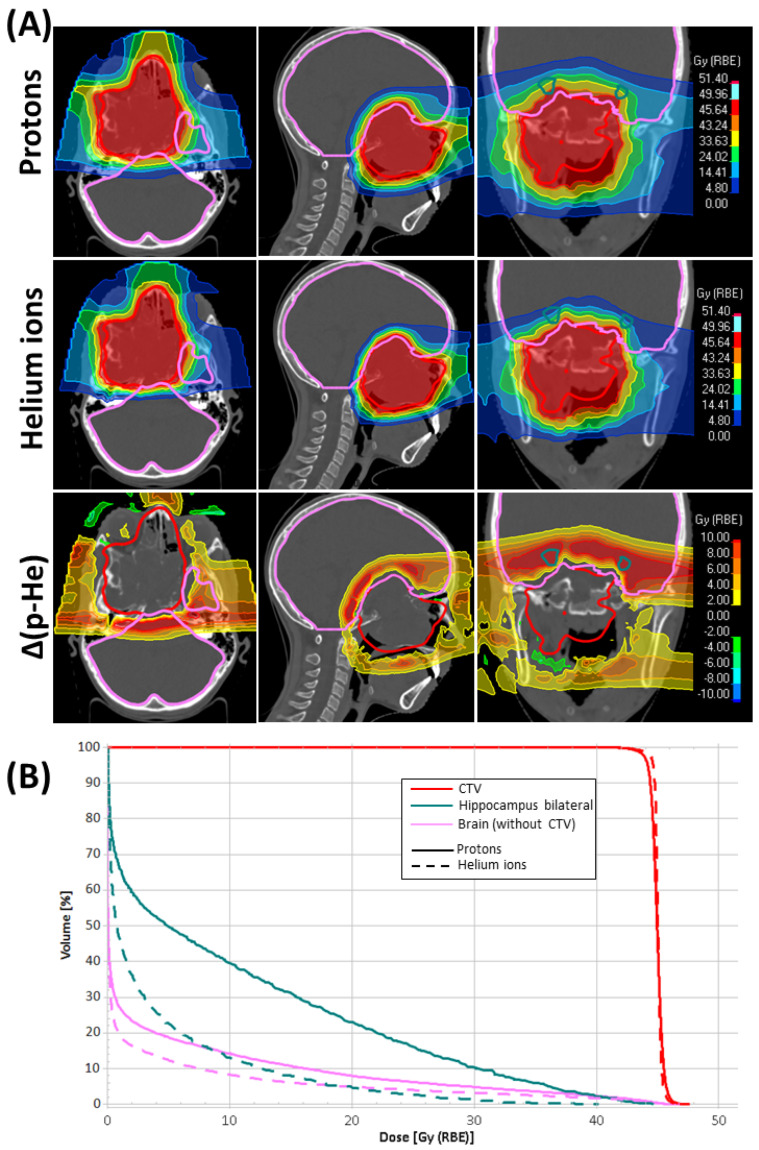
(**A**) Dose distribution on the axial, sagittal and coronal planning CT slices for an exemplary patient with protons and helium ions and the dose difference between the two modalities (Δ(p-He)). (**B**) Dose–volume histograms (DVH) for selected volumes for both modalities. The clinical target volume (CTV) is displayed together with bilateral hippocampus and brain (without the CTV) on the dose maps and DVH. DVH from protons and helium ions plans are represented with solid and dashed lines respectively.

Table 4 and Supplementary Table S2 summarize the absolute and relative differences between the two modalities for the DVH parameters of OAR. Differences in RBE-weighted dose distribution on the planning CT and DVH for CTV and selected OAR are illustrated in Figure 1 for an exemplary patient.

All the investigated NTCP endpoints showed significant differences in favor of helium ions, as displayed in Table 5 and Supplementary Table S3, except for hearing loss, with a 0% probability predicted by both applied models.

Based on the above-mentioned dose reduction to the bilateral hippocampi, the estimated probability of delayed recall was reduced by 3.5% in helium plans, with a relative difference of 25%. Individual patients presented absolute differences above 5% up to approximately 25% regarding delayed recall.

Regarding the probability for skin alopecia, a mean absolute reduction of 6.4% was noted. The mean absolute NTCP differences for the ipsilateral organs outside the brain ranged from 2% to 6%, while relative differences of as much as 30–50% were estimated. The probability of the development of skin erythema as well as NTCP for selected contralateral organs, such as the contralateral cochlea, lens or parotid, showed absolute probability reductions of below 1%. The mean relative risk of radiation-induced secondary CNS malignancies of protons over helium ions was 1.4, with individual risks ranging from 1.1 to about 1.8.

**Figure 2:**
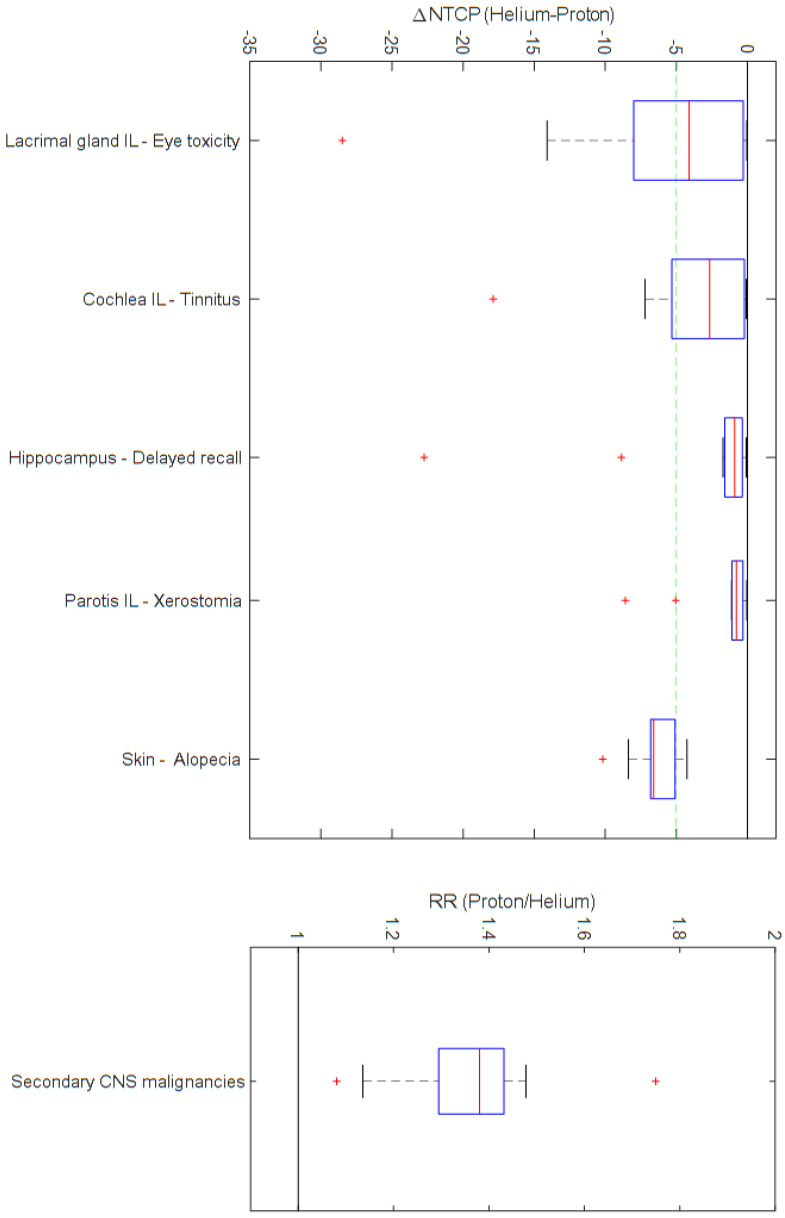
Differences in normal tissue complication probability (∆NTCP) and risk ratio (RR) for secondary CNS malignancies following helium ion therapy and proton therapy. All the presented results are statistically significant. ∆NTCP is expressed in %. IL: ipsilateral.

Differences in NTCP for selected organs at risk regarding the most relevant endpoints and the RR for secondary CNS malignancies following HRT and PRT are presented in Figure 2, Table 5 and in more detail in Supplementary Table S3.

## 4. Discussion

As helium ions are being reintroduced into clinical radiotherapy, ongoing investigations seek to identify which patient cohorts may benefit most from HRT, with pediatric patients emerging as potential candidates [11].

This study presents promising findings regarding the dosimetric advantages of HRT over PRT for advanced JNA, demonstrating enhanced target coverage for all patients and significant reductions in dose deposition in surrounding healthy tissue, thereby potentially mitigating acute and late-term complications.

Dosimetric advantages and subsequent reductions in NTCP were observed across all OAR, with the extent of the individual reductions depending on their proximity to the target volume. The relative differences in dose distribution between the two modalities were generally notable, while the absolute differences may appear relatively small for most OAR since PRT already presents a high degree of conformality [8] and the dose prescription of 45 Gy (RBE) is relatively low.

The radiation dose to OAR critical for neurocognitive function, such as the mean dose to bilateral hippocampi, was reduced by up to 3 Gy (RBE), with a mean dose reduction of 54% in HRT plans compared to PRT. In the hippocampal dentate gyrus are located neural stem cells niches contributing to neurogenesis throughout life and thus playing a key role in learning and memory function [34]. While the exact pathogenesis of radiation-induced neurocognitive impairment is dependent on the interplay of multiple different cell types and their respective microenvironment [35], cognitive sequelae regarding learning and memory function have been correlated with radiation damage to hippocampal structures [36]. This holds particular importance in pediatric patients, as radiation-induced neurocognitive deficiencies are closely related to the patients’ age at the time of treatment [37]. In our study, we observed an estimated absolute reduction of 3.5% in delayed recall for patients receiving HRT compared to PRT. This reduction is not negligible, given notably the long life expectancy of patients affected by JNA and the potential impact of neurocognitive sequelae on their quality of life.

Moreover, improved dosimetric distribution in other OAR beyond the brain translated into reduced estimated risks for certain radiation-induced complications. Specifically, HRT was linked to a lower risk of developing cataracts and notably ocular toxicity, with a mean risk reduction of 6.5%. Additionally, based on reduced dose delivery to the parotid, HRT was associated with a lower probability of xerostomia related to periodontal disease and caries development compared to PRT [38,39]. In line with Wickert et al., who investigated different NTCP following HRT for ependymoma, we found reduced estimates for ipsilateral tinnitus, a condition previously linked to children’s behavioral development [12,40].

With relatively pronounced reductions in integral dose, ranging from approximately 31% for the ipsilateral parotid gland, 26–30% for the supra- and infratentorial brain and 20% for the skin, HRT appears to be a promising modality for alleviating long-term complications such as secondary neoplasms in these regions, as notably low to moderate doses were reduced and previous findings have suggested an association between cancer risks and exposure to such dose levels [41]. The dose reductions observed in our study gain increasing relevance in light of previous analyses of childhood cancer survivors, supporting a linear dose–risk relationship for various secondary neoplasms, including sarcoma, nonmelanoma skin cancer, glioma and salivary gland cancer [41,42]. Among these, the steepest dose–response was reported for meningioma, with an increasing trend in individuals exposed at younger ages [43], emphasizing the relevance of even small absolute differences in dose. By performing risk assessments for radiation-induced secondary CNS malignancies, we found a mean relative risk of 1.4 for PRT over HRT. Taking into account our preliminary investigation comparing PRT and photon radiotherapy within the same cohort, the latter exhibits a relative risk of 2.95 compared to HRT [8]. However, the applied risk assessment model does not account for risks such as the induction of meningioma or the carcinogenic impact of secondary neutrons. Indeed, for a similar treatment dose, protons produce a higher number of ambient neutrons compared to heavier ions [44], thereby potentially further increasing their impact on radiation-induced secondary malignancies. Nevertheless, additional investigations are required to better estimate the neutron dose for the two modalities and its impact on the risk of the induction of secondary neoplasms following HRT or PRT in pediatric patients.

The benefits of HRT may be more pronounced when accounting for potential variations in proton RBE. Indeed, while currently a generic RBE of 1.1 for clinical PRT is recommended, emerging evidence indicates that proton RBE may increase at the distal edge of a proton beam [17], which could accentuate differences in NTCP between the two modalities. Additionally, for HRT planning in this study, an α/β ratio of 2 Gy was applied for all OAR, consistent with the standard clinical practice for helium and carbon ion therapy. This conservative approach is generally preferred in order to overestimate potential OAR toxicity [45]. Implementing a higher α/β ratio for the calculation of RBE-weighted doses would lead to a reduction in the predicted dose, subsequently amplifying dose differences and NTCP between HRT and PRT. In our approach for helium planning, we adhered to the same number of beams and beam angulations employed in clinical proton planning. Typically, in PRT, the utilization of three or more beams aims to counteract the aforementioned potential elevation in RBE at the distal edge of the beams. However, since a variable RBE is inherently considered for helium ions, reducing the number of beams to two, as is commonly practiced in clinical carbon ion planning, becomes feasible. This adjustment has the potential to spare a larger brain volume but may simultaneously heighten the risk in certain OAR.

Ultimately, considering the substantial relative differences between HRT and PRT, patients with tumors requiring higher prescription doses may derive even greater benefit from HRT, warranting further investigation in forthcoming studies.

Despite the promising benefits of HRT for conditions such as advanced JNA, its accessibility remains limited, primarily due to the scarcity of treatment centers that are equipped with the necessary technical infrastructure to provide this therapy. However, advancements in cyclotron technology and compact solutions may pave the way for wider implementation of HRT in the future [46,47].

A major limitation of this study stems from the relatively small patient cohort, which is attributed to the rarity of advanced JNA and is consistent with most previous studies focusing on radiotherapy for advanced JNA. Furthermore, the applied NTCP and risk assessment models were initially developed for different tumor entities, patient demographics and irradiation modalities, underscoring the need for cautious evaluation of the findings.

Nevertheless, the comparisons conducted in this study provide first valuable insights into the potential advantages of HRT for future clinical application in critically located pediatric tumors such as advanced JNA.

## 5. Conclusions

HRT presents a promising treatment modality for advanced JNA, offering enhanced sparing of critical OAR without compromising target coverage compared to PRT. Complication risk assessments suggest that HRT holds the potential to further mitigate both acute and long-term radiation-induced sequelae, including neurocognitive impairment and secondary neoplasms, which is particularly important considering the young age of patients with JNA and their favorable life expectancy.

## Figures and Tables

**Table 1:** Patient, tumor and treatment characteristics. GTV: gross tumor volume.

Patient, Tumor and Treatment Characteristics	
Median patient age	14 (range 12–21) years
Radkowski tumor stage	III
Median GTV volume	35 (range 7–166) cm^3^
Treatment setting	
Definitive setting	*n* = 6 (55%)
Postoperative setting	*n* = 5 (45%)
Number of treatment beams	
Two beams	*n* = 3 (27%)
Three beams	*n* = 5 (46%)
Four beams	*n* = 3 (27%)
Treatment room	
Isocentric gantry	*n* = 3 (27%)
Fixed 90° beam line	*n* = 8 (73%)

**Table 2:** Models used for normal tissue complication probability (NTCP) and intellectual quotient (IQ) estimations following radiotherapy. TD_50_: dose leading to a complication probability of 50%; gEUD: generalized equivalent uniform dose; IL: ipsilateral; CL: contralateral; ACTH: adrenocorticotropic hormone; GH: growth hormone.

Organ at Risk	Complication	Formula	Parameters	Publication
Brain	IQ estimation (Wechsler Intelligence Scale)	$\begin{array}{cc} IQ & =\\ 93.11+time\;\times & (0.028\times age\\ & -\;0.0095\\ & \times \;Dmean) \end{array}$	time = time since radiotherapy (set at 5 years) age = age of the patient at the time of radiotherapy D_mean_ = average dose	Merchant 2006 [21]
	IQ estimation at a long follow-up time	$\begin{array}{cc} IQ & =\\ 108.1-Dmean & \times \;(0.61593\\ & -\;0.02328\\ & \times \;age) \end{array}$	age = age of the patient at the time of radiotherapy D_mean_ = average dose in 2 Gy (RBE) equivalent dose using an α/β = 3 Gy	Mahajan 2021 [22]
	Neurocognitive impairment estimation at a long follow-up time (IQ < 85)	NTCP=12π ∫−∞te−x22dxt=D − TD50m × TD50D=gEUD=[∑i(viDi1n)]n	a = 3.39 n = 1/a TD_50_ = 33.5 Gy (RBE) m = 0.28 α/β = 3 Gy v_i_, corresponding to a volume fraction of a structure receiving a dose D_i_ in 2 Gy (RBE) equivalent dose using an α/β = 3 Gy	Mahajan 2021 [22]
Infratentorial brain	IQ estimation (Wechsler Intelligence Scale)	$\begin{array}{cc} IQ & =\\ 93.23+time\;\times & (0.034\times age\\ & -\;0.0077\\ & \times \;Dmean) \end{array}$	time = time since radiotherapy (set at 5 years) age = age of the patient at the time of radiotherapy D_mean_ = average dose	Merchant 2006 [21]
Supratentorial brain	IQ estimation (Wechsler Intelligence Scale)	$\begin{array}{cc} IQ & =\\ 93.00+time\;\times & (0.024\times age\\ & -\;0.0091\\ & \times \;Dmean) \end{array}$	time = time since radiotherapy (set at 5 years) age = age of the patient at the time of radiotherapy D_mean_ = average dose	Merchant 2006 [21]
Cochlea (IL/CL)	Tinnitus (grade ≥ 2, 1–2 years post radiotherapy)	NTCP=12π ∫−∞te−x22dxt=D − TD50m × TD50D=gEUD=[∑i(viDi1n)]n	n = 1.0 TD_50_ = 46.52 Gy (RBE) m = 0.35 α/β = 3.25 Gy v_i_, corresponding to a volume fraction of a structure receiving a dose D_i_ in 2 Gy equivalent dose using an α/β = 3.25 Gy	Dell’Oro 2021 [25] adapted from Lee 2015 [29] and De Marzi 2015 [30]
	Hearing loss (grade ≥ 1–2, 2 years post radiotherapy)	NTCP=12π ∫−∞te−x22dxt=D − TD50m × TD50D=gEUD=[∑i(viDi1n)]n	n = 1.0 TD_50_ = 55.57 Gy (RBE) m = 0.14 α/β = 3.25 Gy v_i_, corresponding to a volume fraction of a structure receiving a dose D_i_ in 2 Gy (RBE) equivalent dose using an α/β = 3.25 Gy	
Hippocampus bilateral	Delayed recall (Wechsler Memory Scale III Word Lists, 1.5 years post radiotherapy)	$\begin{array}{c} NTCP=\frac{1}{\sqrt{2\pi }}\;\int_{-\infty }^{t}e^{\frac{-x^{2}}{2}}dx\\ t=\frac{D\;-\;{TD}_{50}}{{m\;\times \;TD}_{50}}\\ D=D40\% \end{array}$	D_40%_ = minimum dose received in 40% of the structure volume, in 2 Gy (RBE) equivalent dose using an α/β = 2.0 Gy TD_50_ = 14.88 Gy (RBE) m = 0.54	Gondi 2012 [23]
Pituitary	Endocrine dysfunction (grade ≥ 1–2, 2 years post radiotherapy)	NTCP=12π ∫−∞te−x22dxt=D − TD50m × TD50D=gEUD=[∑i(viDi1n)]n	n = 0.25 TD_50_ = 60.6 Gy (RBE) m = 0.15 α/β = 2.5 Gy v_i_, corresponding to a volume fraction of a structure receiving a dose D_i_ in 2 Gy (RBE) equivalent dose using an α/β = 2.5 Gy	Dell’Orro 2021 [25] adapted from De Marzi 2015 [30]
	GH deficiency (5 years post radiotherapy)	$NTCP={\left(1+e^{4\gamma \times \;(1-\frac{D2\%}{{TD}_{50}})}\right)}^{-1}$	TD_50_ = 27.2 Gy (RBE) γ = 0.5 D_2%_ = minimum dose received in 2% of the structure, in 2 Gy (RBE) equivalent dose using an α/β = 3 Gy	Wheeler 2023 [24]
	Hypothyroidism (5 years post radiotherapy)	$NTCP={\left(1+e^{4\gamma \times \;(1-\frac{D2\%}{{TD}_{50}})}\right)}^{-1}$	TD_50_ = 39.2 Gy (RBE) γ = 0.75 D_2%_ = minimum dose received in 2% of the structure, in 2 Gy (RBE) equivalent dose using an α/β = 3 Gy	
	ACTH-deficiency (5 years post radiotherapy)	$NTCP={\left(1+e^{4\gamma \times \;(1-\frac{D2\%}{{TD}_{50}})}\right)}^{-1}$	TD_50_ = 58.0 Gy (RBE) γ = 0.74 D_2%_ = minimum dose received in 2% of the structure, in 2 Gy (RBE) equivalent dose using an α/β = 3 Gy	
Lacrimal gland IL	Ocular toxicity (grade ≥ 2, acute toxicity)	$NTCP={\left(1+e^{-\beta 0-\beta 1\times Dmax)}\right)}^{-1}$	D_max_ = minimum dose received in 0.03 cm^3^ of the structure β_0_ = −5.174 β_1_ = 0.205 Gy^−1^	Batth 2013 [26]
Lens (IL/CL)	Cataract (5 years post radiotherapy)	NTCP=12π ∫−∞te−x22dxt=D − TD50m × TD50D=gEUD=[∑i(viDi1n)]n	a = 3.33 n = 1/a TD_50_ = 18.0 Gy (RBE) m = 0.27 v_i_, corresponding to a volume fraction of a structure receiving a dose D_i_	Burman 1991 [31]
Parotis (IL/CL)	Xerostomia (1 year post radiotherapy	NTCP=12π ∫−∞te−x22dxt=D − TD50m × TD50D=gEUD=[∑i(viDi1n)]n	n = 1.0 TD_50_ = 39.9 Gy (RBE) m = 0.4 v_i_, corresponding to a volume fraction of a structure receiving a dose D_i_	Houweling 2010 [28]
Skin	Alopecia (grade ≥ 2, acute toxicity)	$NTCP={\left(1+e^{-\beta 0-\beta 1\times D5\%)}\right)}^{-1}$	D_5%_ = minimum dose received in 5% of the volume structure β_0_ = −1.33 β_1_ = 0.08 Gy^−1^	Dutz 2019 [27]
	Erythema (grade ≥ 2, acute toxicity)	$NTCP={\left(1+e^{-\beta 0-\beta 1\times V35)}\right)}^{-1}$	V_35_ = volume of the structure in cm^3^ receiving a minimum dose of 35 Gy (RBE) β_0_ = −1.54 β_1_ = 0.06 cm^−3^	

**Table 3:** Dosimetric parameters regarding the clinical target volume (CTV). Doses D_x_ are expressed in percentage of the prescribed dose and volumes in % of the structure volume. Reported values are rounded to one decimal place. V_x_: volume receiving x% of the prescribed dose; D_x_: dose delivered to x% or x cm^3^ of the CTV; HI: homogeneity index; CI: conformity index; SD: standard deviation; ∆abs: absolute difference; ∆rel: relative difference in %. Additional information can be found in Supplementary Table S1.

		Helium			Proton			Δabs (Helium–Proton)			Δrel (Helium–Proton)			
		Mean	±	SD	Mean	±	SD	Mean	±	SD	Mean	±	SD	*p*-Value
CTV	D_0.03cm^3^_	103.6	±	0.6	105.3	±	2.2	−1.7	±	1.8	−1.6	±	1.7	0.001
	D_2%_	101.8	±	0.5	102.8	±	0.6	−1.1	±	0.5	−1.0	±	0.5	0.001
	D_95%_	98.4	±	1.2	97.2	±	1.8	1.2	±	0.8	1.2	±	0.9	0.003
	V_95%_	99.4	±	1.3	98.0	±	4.2	1.4	±	3.0	1.6	±	3.5	0.020
	V_107%_	0.0	±	0.0	0.1	±	0.2	−0.1	±	0.2	-	±	-	0.016
	HI	2.9	±	1.3	5.0	±	1.8	−2.1	±	0.8	−42.9	±	11.2	0.001
	CI	0.57	±	0.19	0.57	±	0.18	0.01	±	0.04	0.3	±	6.0	1.000

**Table 4:** Selected dosimetric parameters related to various organs at risk (OAR). Doses D_x_ are expressed in Gy (RBE), integral dose (ID) is expressed in cm^3^ x Gy (RBE) and volumes in % of the structure volume, except for skin volumes, which are expressed in cm^3^. Reported values are rounded to one decimal place. V_x_: volume of the OAR receiving a minimum dose of x Gy (RBE); D_0.03cm^3^_: minimum dose received in 0.03 cm^3^ of the OAR; D_mean_: mean dose received by the OAR; CTV: clinical target volume; CL: contralateral; IL: ipsilateral, SD: standard deviation; ∆abs: absolute difference; ∆rel: relative difference in %. Additional information can be found in Supplementary Table S2.

		Helium			Proton			Δabs (Helium–Proton)			Δrel (Helium–Proton)			
		Mean	±	SD	Mean	±	SD	Mean	±	SD	Mean	±	SD	*p*-Value
Brain (without CTV)	D_0.03cm^3^_	45.1	±	0.8	45.4	±	0.9	−0.3	±	0.4	−0.6	±	0.9	0.039
	D_mean_	2.2	±	1.1	2.9	±	1.4	−0.8	±	0.5	−26.5	±	9.5	0.001
	V_10_	6.8	±	3.6	11.1	±	6.5	−4.3	±	3.9	−35.1	±	16.2	0.001
	V_15_	4.4	±	2.3	7.0	±	3.8	−2.6	±	2.0	−34.6	±	13.8	0.001
	V_20_	3.0	±	1.6	4.6	±	2.5	−1.5	±	1.0	−32.5	±	9.4	0.001
	ID	3538.1	±	1815.5	4812.2	±	2337.4	−1274.1	±	828.4				
Brain supratentorial	D_0.03cm^3^_	45.5	±	0.5	46.0	±	0.6	−0.6	±	0.4	−1.1	±	0.8	0.003
	D_mean_	2.4	±	1.2	3.3	±	1.6	−0.9	±	0.8	−25.7	±	1.8	0.001
	V_10_	7.8	±	4.4	12.2	±	7.3	−4.4	±	4.2	−32.8	±	14.0	0.001
	V_15_	5.1	±	2.8	7.7	±	4.3	−2.6	±	2.1	−31.5	±	8.0	0.001
	V_20_	3.7	±	2.1	5.1	±	2.9	−1.5	±	1.0	−27.9	±	16.8	0.001
	ID	3409.0	±	1839.4	4619.1	±	2391.3	−1210.1	±	924.3				
Brain infratentorial	D_0.03cm^3^_	43.0	±	6.5	43.3	±	6.5	−0.4	±	0.6	−0.8	±	4.9	0.067
	D_mean_	1.9	±	1.1	2.6	±	1.4	−0.8	±	0.5	−29.5	±	8.9	0.001
	V_10_	5.3	±	3.2	8.0	±	4.4	−2.7	±	1.6	−36.0	±	6.3	0.001
	V_15_	4.2	±	2.8	6.2	±	3.8	−2.0	±	1.3	−35.0	±	12.2	0.001
	V_20_	3.3	±	2.5	4.8	±	3.3	−1.5	±	1.1	−33.4	±	21.7	0.001
	ID	472.0	±	289.0	657.0	±	343.2	−185.0	±	102.5				
Brainstem	D_0.03cm^3^_	32.9	±	10.3	35.8	±	8.7	−2.9	±	2.7	−9.6	±	9.9	0.002
	D_mean_	4.8	±	3.9	7.7	±	4.8	−2.9	±	1.7	−41.7	±	15.2	0.001
	ID	134.4	±	108.7	213.5	±	137.1	−79.1	±	50.4				
Hippocampus bilateral	D_0.03cm^3^_	20.9	±	13.0	28.8	±	11.6	−7.9	±	4.5	−32.7	±	17.8	0.001
	D_40%_	1.6	±	3.1	4.0	±	5.3	−2.4	±	2.7	−65.7	±	14.7	0.001
	D_mean_	2.8	±	2.7	5.6	±	3.9	−2.8	±	1.8	−54.1	±	14.4	0.001
	ID	11.4	±	10.0	23.1	±	14.5	−11.6	±	7.6				
Hippocampus CL	D_0.03cm^3^_	12.5	±	13.9	18.7	±	15.4	−6.2	±	4.2	−50.3	±	25.7	0.001
	D_mean_	1.8	±	2.2	3.7	±	3.9	−1.9	±	2.1	−57.9	±	15.8	0.001
	ID	3.7	±	4.4	7.3	±	7.2	−3.7	±	3.9				
Hippocampus IL	D_0.03cm^3^_	19.9	±	13.6	27.9	±	12.1	−8.0	±	5.0	−35.2	±	20.4	0.001
	D_mean_	3.9	±	3.5	7.7	±	4.9	−3.8	±	2.4	−53.1	±	14.8	0.001
	ID	7.8	±	6.2	15.7	±	9.3	−8.0	±	5.2				
Pituitary	D_0.03cm^3^_	39.2	±	8.1	41.1	±	6.5	−1.8	±	2.2	−5.2	±	7.6	0.001
	D_mean_	36.3	±	11.4	37.9	±	10.1	−1.7	±	1.7	−6.1	±	8.8	0.003
	ID	14.6	±	7.3	15.3	±	7.2	−0.7	±	0.6				
Chiasma	D_0.03cm^3^_	36.5	±	12.3	37.6	±	11.0	−1.1	±	1.4	−5.3	±	9.0	0.007
	D_mean_	25.2	±	15.0	27.0	±	14.2	−1.7	±	2.1	−11.5	±	12.7	0.024
	ID	38.7	±	26.6	41.8	±	28.2	−3.1	±	5.1				
Optic nerve CL	D_0.03cm^3^_	36.3	±	10.8	36.9	±	10.4	−0.6	±	1.2	−2.2	±	4.5	0.102
	D_mean_	26.2	±	13.3	27.8	±	12.3	−1.6	±	2.0	−9.2	±	12.2	0.010
	ID	30.6	±	33.0	31.8	±	32.1	−1.2	±	1.9				
Optic nerve IL	D_0.03cm^3^_	41.8	±	2.7	41.9	±	2.9	−0.2	±	0.6	−0.4	±	1.5	0.377
	D_mean_	34.4	±	9.0	35.4	±	8.0	−1.0	±	1.2	−3.6	±	4.6	0.054
	ID	35.1	±	28.0	35.8	±	27.2	−0.7	±	1.1				
Eye CL	D_0.03cm^3^_	14.2	±	10.5	19.1	±	9.0	−4.9	±	4.1	−32.0	±	29.8	0.001
	D_mean_	4.0	±	3.6	6.1	±	4.3	−2.1	±	1.6	−43.0	±	25.4	0.001
	V_10_	11.1	±	15.6	19.1	±	23.4	−8.0	±	9.8	−66.7	±	31.4	0.001
	ID	36.9	±	33.5	56.4	±	41.3	−19.5	±	15.6				
Eye IL	D_0.03cm^3^_	26.9	±	12.4	28.6	±	11.3	−1.6	±	3.5	−8.3	±	17.3	0.123
	D_mean_	9.4	±	6.8	11.1	±	7.2	−1.8	±	1.3	−22.3	±	17.9	0.001
	V_10_	36.6	±	28.5	43.1	±	30.2	−7.5	±	5.9	−35.5	±	34.8	0.001
	ID	87.2	±	65.0	103.4	±	69.4	−16.2	±	12.0				
Lens CL	D_0.03cm^3^_	2.3	±	2.1	4.2	±	3.6	−1.9	±	2.2	−47.6	±	25.1	0.001
	D_mean_	1.7	±	1.8	3.2	±	2.8	−1.5	±	1.8	−47.9	±	27.7	0.001
	ID	0.3	±	0.3	0.7	±	0.6	−0.3	±	0.4				
Lens IL	D_0.03cm^3^_	4.9	±	5.8	6.8	±	7.3	−0.1	±	1.9	−37.5	±	20.2	0.001
	D_mean_	3.6	±	4.2	5.3	±	5.3	−1.6	±	1.5	−39.8	±	18.8	0.001
	ID	0.8	±	1.0	1.1	±	1.4	−0.4	±	0.4				
Lacrimal gland CL	D_0.03cm^3^_	3.6	±	4.0	5.5	±	4.8	−1.9	±	1.8	−41.6	±	28.5	0.001
	D_mean_	1.8	±	2.3	3.0	±	3.6	−1.2	±	1.7	−40.1	±	37.5	0.007
	ID	1.9	±	3.0	3.3	±	5.5	−1.4	±	2.6				
Lacrimal gland IL	D_0.03cm^3^_	10.1	±	8.9	13.2	±	10.4	−3.1	±	2.7	−28.2	±	18.3	0.001
	D_mean_	5.2	±	4.9	7.1	±	6.3	−1.9	±	1.8	−31.3	±	16.8	0.001
	ID	4.5	±	5.3	6.3	±	7.3	−1.8	±	2.1				
Cochlea CL	D_0.03cm^3^_	4.9	±	6.1	8.1	±	6.7	−3.3	±	2.6	−52.6	±	24.1	0.002
	D_mean_	4.1	±	5.4	7.3	±	6.1	−3.1	±	2.5	−55.3	±	23.5	0.002
	ID	0.5	±	0.7	0.9	±	0.8	−0.4	±	0.3				
Cochlea IL	D_0.03cm^3^_	17.1	±	12.0	22.7	±	13.7	−5.6	±	4.5	−29.5	±	17.2	0.001
	D_mean_	14.7	±	10.3	20.7	±	12.4	−5.9	±	4.3	−32.9	±	16.5	0.001
	ID	2.1	±	2.2	2.8	±	2.6	−0.8	±	0.7				
Parotis CL	D_0.03cm^3^_	11.3	±	5.5	16.0	±	6.4	−4.7	±	3.8	−29.5	±	18.0	0.002
	D_mean_	3.9	±	3.6	6.8	±	4.1	−2.8	±	2.2	−45.4	±	23.6	0.002
	ID	74.7	±	74.9	125.1	±	93.7	−50.4	±	45.3				
Parotis IL	D_0.03cm^3^_	23.1	±	13.1	26.9	±	11.9	−3.8	±	2.8	−17.9	±	13.3	0.001
	D_mean_	8.1	±	7.9	10.6	±	8.9	−2.5	±	1.8	−31.4	±	15.7	0.001
	ID	120.5	±	109.8	159.4	±	122.8	−38.9	±	25.9				
Skin	D_0.03cm^3^_	32.2	±	10.7	34.0	±	9.3	−1.8	±	2.4	−6.7	±	9.0	0.019
	V_10_	32.4	±	27.9	49.4	±	30.9	−17.0	±	5.8	−31.2	±	6.3	0.001
	V_15_	15.3	±	15.7	27.5	±	26.5	−12.2	±	11.9	−41.0	±	15.4	0.001
	V_20_	7.6	±	8.5	13.2	±	12.7	−5.6	±	5.0	−51.7	±	21.6	0.002
	ID	876.4	±	51.5.5	1235.5	±	624.1	−359.1	±	118.2				

**Table 5:** Normal tissue complication probability (NTCP) for selected organs at risk, and the risk ratio (RR) for secondary CNS malignancies. Reported values are rounded to one decimal place. NTCP is expressed in %. IL: ipsilateral; SD: standard deviation; ∆abs: absolute difference. Additional information can be found in Supplementary Table S3.

		Helium			Proton			Δabs (Helium–Proton)			
Organ at Risk	Complication	Mean	±	SD	Mean	±	SD	Mean	±	SD	*p*-Value
Cochlea IL	Tinnitus	3.0	±	3.6	7.0	±	7.6	−4.0	±	5.3	0.001
Hippocampus (bilateral)	Delayed recall	4.6	±	3.5	8.0	±	10.2	−3.5	±	6.9	0.001
Lacrimal gland IL	Ocular toxicity	11.8	±	18.5	18.1	±	27.0	−6.3	±	8.6	0.001
Lens IL	Cataract	3.4	±	10.5	7.4	±	22.9	−3.9	±	12.9	0.001
Parotis IL	Xerostomia	3.9	±	5.5	5.6	±	7.6	−1.7	±	2.7	0.001
Skin	Alopecia	38.0	±	9.4	44.5	±	10.2	−6.4	±	1.7	0.001
		RR (Proton/Helium)									
		Mean	±	SD	*p*-value						
Brain	Secondary malignancies	1.4	±	0.2	0.001						

## Data Availability

The data presented in this study are available on request from the corresponding author.

## Supplementary Materials

The following supporting information can be downloaded at https://www.mdpi.com/article/10.3390/cancers16111993/s1, Table S1: Dosimetric parameters regarding the clinical target volume; Table S2: Dosimetric parameters related to various organs at risk; Table S3: Normal tissue complication probability for selected organs at risk, and the risk ratio for secondary CNS malignancies.
