# Supplementary material for: Helium Ion Therapy for Advanced Juvenile Nasopharyngeal Angiofibroma
Source: Cancers (Basel). 2024 May 24;16(11):1993. doi: 10.3390/cancers16111993 (PMC11171253; doi:10.3390/cancers16111993)
Supplement: Supplementary file 1 [file cancers-16-01993-s001.zip › Supplementary Table S1.pdf]

Supplementary Table S1: Dosimetric parameters regarding the clinical target volume (CTV). Doses D_x are expressed in percentage of the prescribed dose and volumes in % of the structure volume. V_x: volume receiving x% of the prescribed dose, D_x: dose delivered to x% or x cm^3 of the CTV. HI: homogeneity index; CI: conformity index; SD: standard deviation; Δabs: absolute difference; Δrel: relative difference in %.

		Helium			Proton			Δabs(Helium - Proton)			Δrel(Helium - Proton)			
		Mean		SD	Mean		SD	Mean		SD	Mean		SD	p-value
CTV	$D_{0.03cm^3}$	103.6	±	0.6	105.3	±	2.2	-1.7	±	1.8	-1.6	±	1.7	0.001
	$D_{2\%}$	101.8	±	0.5	102.8	±	0.6	-1.1	±	0.5	-1.0	±	0.5	0.001
	$D_{5\%}$	101.2	±	0.4	102.2	±	0.5	-0.9	±	0.4	-0.9	±	0.4	0.002
	$D_{95\%}$	98.4	±	1.2	97.2	±	1.8	1.2	±	0.8	1.2	±	0.9	0.003
	$D_{98\%}$	97.6	±	1.5	96.1	±	2.3	1.5	±	1.0	1.6	±	1.1	0.001
	$V_{95\%}$	99.4	±	1.3	98.0	±	4.2	1.4	±	3.0	1.6	±	3.5	0.020
	$V_{105\%}$	0.0	±	0.0	0.1	±	0.1	-0.2	±	0.4	-	±	-	0.125
	$V_{107\%}$	0.0	±	0.0	0.1	±	0.2	-0.1	±	0.2	-	±	-	0.016
	HI	2.9	±	1.3	5.0	±	1.8	-2.1	±	0.8	-42.9	±	11.2	0.001
	CI	0.57	±	0.19	0.57	±	0.18	0.01	±	0.04	0.3	±	6.0	1.000
