# Supplementary material for: Helium Ion Therapy for Advanced Juvenile Nasopharyngeal Angiofibroma
Source: Cancers (Basel). 2024 May 24;16(11):1993. doi: 10.3390/cancers16111993 (PMC11171253; doi:10.3390/cancers16111993)
Supplement: Supplementary file 1 [file cancers-16-01993-s001.zip › Supplementary Table S2.pdf]

Supplementary Table S2: Selected dosimetric parameters related to various organs at risk (OAR). Doses D_x are expressed in Gy (RBE), integral dose (ID) is expressed in cm^3 x Gy (RBE) and volumes in % or cm^3 of the structure volume. V_x: volume of the OAR receiving a minimum dose of x Gy (RBE), Dx: minimum dose received in x % of the OAR, $D_{0.03\ cm^3}$: minimum dose received in 0.03 cm^3 of the OAR and D_{mean}: mean dose received by the OAR. CTV: clinical target volume; CL: contralateral; IL: ipsilateral, SD: standard deviation; Δabs: absolute difference; Δrel: relative difference in %.

		Helium			Proton			Δ_{abs}(Helium - Proton)			Δ_{rel}(Helium - Proton)			
		Mean	±	SD	Mean	±	SD	Mean	±	SD	Mean	±	SD	p-value
Brain	$D_{0.03cm^3}$	45.5	±	0.6	46.0	±	0.7	-0.5	±	0.4	-1.0	±	0.8	0.003
	$D_{2\%}$	29.1	±	12.8	31.9	±	11.1	-2.9	±	2.5	-12.0	±	11.0	0.007
	D_{mean}	2.4	±	1.2	3.2	±	1.5	-0.8	±	0.6	-25.7	±	9.7	0.001
	$V_{20}(cm^3)$	61.1	±	40.7	86.0	±	54.5	-24.9	±	16.7	-29.2	±	8.7	0.001
	$V_{20}(\%)$	3.6	±	2.1	5.1	±	2.9	-1.5	±	1.0				
	ID	3933.1	±	2132.4	5270.4	±	2650.1	-1337.3	±	894.5				
Brain (without CTV)	$D_{0.03cm^3}$	45.1	±	0.8	45.4	±	0.9	-0.3	±	0.4	-0.6	±	0.9	0.039
	$D_{2\%}$	26.1	±	10.7	29.5	±	9.5	-3.4	±	2.2	-14.1	±	10.3	0.001
	$D_{50\%}$	0.1	±	0.1	0.1	±	0.1	0.0	±	0.1	-	±	-	0.231
	D_{mean}	2.2	±	1.1	2.9	±	1.4	-0.8	±	0.5	-26.5	±	9.5	0.001
	$V_{10}(cm^3)$	111.9	±	61.9	181.9	±	107.1	-70.0	±	63.3	-35.1	±	16.2	0.001
	$V_{15}(cm^3)$	72.6	±	42.4	114.9	±	66.3	-42.3	±	33.4	-34.6	±	13.8	0.001
	$V_{20}(cm^3)$	50.4	±	30.0	75.8	±	44.7	-25.4	±	17.2	-32.5	±	9.4	0.001
	$V_{35}(cm^3)$	22.3	±	17.0	27.5	±	19.5	-5.2	±	4.6	-21.1	±	9.4	0.001
	$V_{10}(\%)$	6.8	±	3.6	11.1	±	6.5	-4.3	±	3.9				
	$V_{15}(\%)$	4.4	±	2.3	7.0	±	3.8	-2.6	±	2.0				
	$V_{20}(\%)$	3.0	±	1.6	4.6	±	2.5	-1.5	±	1.0				
	$V_{35}(\%)$	1.3	±	0.9	1.7	±	1.2	-0.3	±	0.3				
	ID	3538.1	±	1815.5	4812.2	±	2337.4	-1274.1	±	828.4				
Brain supratentorial	$D_{0.03cm^3}$	45.5	±	0.5	46.0	±	0.6	-0.6	±	0.4	-1.1	±	0.8	0.003
	$D_{2\%}$	29.4	±	12.9	32.0	±	11.2	-2.6	±	2.6	-10.9	±	10.5	0.014
	$D_{50\%}$	0.1	±	0.1	0.1	±	0.1	0.0	±	0.1	-	±	-	0.410
	D_{mean}	2.4	±	1.2	3.3	±	1.6	-0.9	±	0.8	-25.7	±	1.8	0.001
	$V_{10}(cm^3)$	110.7	±	66.5	172.3	±	105.3	-61.6	±	57.9	-32.8	±	14.0	0.001
	$V_{15}(cm^3)$	73.7	±	47.2	109.9	±	67.1	-36.2	±	30.0	-31.5	±	8.0	0.001
	$V_{20}(cm^3)$	53.1	±	34.9	74.0	±	47.8	-20.9	±	14.8	-27.9	±	16.8	0.001
	$V_{10}(\%)$	7.8	±	4.4	12.2	±	7.3	-4.4	±	4.2				
	$V_{15}(\%)$	5.1	±	2.8	7.7	±	4.3	-2.6	±	2.1				
	$V_{20}(\%)$	3.7	±	2.1	5.1	±	2.9	-1.5	±	1.0				
	ID	3409.0	±	1839.4	4619.1	±	2391.3	-1210.1	±	924.3				
Brain infratentorial	$D_{0.03cm^3}$	43.0	±	6.5	43.3	±	6.5	-0.4	±	0.6	-0.8	±	4.9	0.067
	$D_{2\%}$	26.4	±	14.1	30.6	±	12.2	-4.3	±	3.3	-18.6	±	20.3	0.002
	$D_{50\%}$	0.1	±	0.1	0.1	±	0.0	0.1	±	0.1	-	±	-	0.008
	D_{mean}	1.9	±	1.1	2.6	±	1.4	-0.8	±	0.5	-29.5	±	8.9	0.001
	$V_{10}(cm^3)$	13.4	±	8.4	20.1	±	10.5	-6.6	±	3.6	-36.0	±	6.3	0.001
	$V_{15}(cm^3)$	10.5	±	7.3	15.4	±	9.1	-4.9	±	3.0	-35.0	±	12.2	0.001

	V_{20}(cm^3)	8.4 ± 6.4	12.1 ± 7.8	-3.7 ± 2.6	-33.4 ± 21.7	0.001
	V_{10}(%)	5.3 ± 3.2	8.0 ± 4.4	-2.7 ± 1.6		
	V_{15}(%)	4.2 ± 2.8	6.2 ± 3.8	-2.0 ± 1.3		
	V_{20}(%)	3.3 ± 2.5	4.8 ± 3.3	-1.5 ± 1.1		
	ID	472.0 ± 289.0	657.0 ± 343.2	-185.0 ± 102.5		
Cerebellum	$D_{0.03cm^3}$	6.7 ± 7.4	12.6 ± 10.7	-5.9 ± 5.4	-52.5 ± 23.1	0.001
	$D_{2\%}$	1.2 ± 1.1	2.8 ± 2.8	-1.7 ± 1.9	-54.0 ± 15.7	0.001
	$D_{50\%}$	0.1 ± 0.1	0.0 ± 0.0	0.1 ± 0.1	- ± -	0.001
	D_{mean}	0.2 ± 0.1	0.3 ± 0.2	-0.1 ± 0.1	-14.9 ± 42.6	0.046
	V_{10}(cm^3)	0.1 ± 0.2	0.5 ± 0.8	-0.4 ± 0.7	-39.1 ± 39.4	0.031
	V_{15}(cm^3)	0.0 ± 0.1	0.2 ± 0.4	-0.2 ± 0.3	-29.1 ± 42.0	0.125
	V_{20}(cm^3)	0.0 ± 0.0	0.1 ± 0.2	-0.1 ± 0.2	-23.6 ± 40.6	0.250
	V_{10}(%)	0.1 ± 0.1	0.3 ± 0.6	-0.3 ± 0.5		
	V_{15}(%)	0.0 ± 0.0	0.1 ± 0.3	-0.1 ± 0.2		
	V_{20}(%)	0.0 ± 0.0	0.1 ± 0.1	-0.1 ± 0.1		
	ID	28.3 ± 19.8	40.3 ± 31.5	-12.0 ± 18.5		
Cerebellum anterior	$D_{0.03cm^3}$	3.6 ± 4.5	8.5 ± 9.1	-5.0 ± 4.9	-56.8 ± 17.8	0.001
	$D_{2\%}$	1.3 ± 1.5	3.4 ± 4.1	-2.1 ± 2.6	-54.8 ± 19.9	0.001
	$D_{50\%}$	0.1 ± 0.1	0.1 ± 0.1	0.0 ± 0.1	- ± -	0.082
	D_{mean}	0.2 ± 0.2	0.4 ± 0.4	-0.2 ± 0.3	-17.2 ± 44.2	0.041
	V_{10}(cm^3)	0.0 ± 0.0	0.1 ± 0.1	-0.1 ± 0.1	-40.5 ± 46.7	0.063
	V_{15}(cm^3)	0.0 ± 0.0	0.0 ± 0.1	0.0 ± 0.1	-23.5 ± 40.5	0.250
	V_{20}(cm^3)	0.0 ± 0.0	0.0 ± 0.0	0.0 ± 0.0	-16.8 ± 37.5	0.500
	V_{10}(%)	0.1 ± 0.2	0.5 ± 1.1	-0.4 ± 0.9		
	V_{15}(%)	0.0 ± 0.1	0.2 ± 0.5	-0.2 ± 0.5		
	V_{20}(%)	0.0 ± 0.0	0.1 ± 0.3	-0.1 ± 0.3		
	ID	3.7 ± 2.2	5.9 ± 5.1	-2.2 ± 3.4		
Cerebellum posterior	$D_{0.03cm^3}$	6.2 ± 7.4	11.1 ± 9.6	-4.9 ± 4.3	-51.3 ± 23.8	0.002
	$D_{2\%}$	1.2 ± 1.2	2.8 ± 3.0	-1.6 ± 1.9	-52.6 ± 16.3	0.001
	$D_{50\%}$	0.1 ± 0.1	0.0 ± 0.0	0.1 ± 0.1	- ± -	0.001
	D_{mean}	0.2 ± 0.1	0.3 ± 0.2	-0.1 ± 0.1	-11.9 ± 45.5	0.079
	V_{10}(cm^3)	0.1 ± 0.2	0.4 ± 0.8	-0.3 ± 0.6	-35.1 ± 41.7	0.063
	V_{15}(cm^3)	0.0 ± 0.1	0.2 ± 0.4	-0.1 ± 0.3	-37.9 ± 40.7	0.125
	V_{20}(cm^3)	0.0 ± 0.0	0.1 ± 0.2	-0.1 ± 0.2	-19.1 ± 34.2	0.250
	V_{10}(%)	0.1 ± 0.1	0.3 ± 0.7	-0.2 ± 0.5		
	V_{15}(%)	0.0 ± 0.0	0.1 ± 0.3	-0.1 ± 0.3		
	V_{20}(%)	0.0 ± 0.0	0.1 ± 0.1	0.0 ± 0.1		
	ID	24.6 ± 18.1	34.3 ± 28.5	-9.7 ± 16.4		
Brainstem	$D_{0.03cm^3}$	32.9 ± 10.3	35.8 ± 8.7	-2.9 ± 2.7	-9.6 ± 9.9	0.002
	$D_{2\%}$	25.2 ± 13.1	30.0 ± 11.1	-4.9 ± 3.3	-20.6 ± 17.2	0.001
	D_{mean}	4.8 ± 3.9	7.7 ± 4.8	-2.9 ± 1.7	-41.7 ± 15.2	0.001
	ID	134.4 ± 108.7	213.5 ± 137.1	-79.1 ± 50.4		
Hippocampus bilateral	$D_{0.03cm^3}$	20.9 ± 13.0	28.8 ± 11.6	-7.9 ± 4.5	-32.7 ± 17.8	0.001
	$D_{2\%}$	17.8 ± 12.5	26.3 ± 11.7	-8.5 ± 4.7	-38.4 ± 19.1	0.001
	$D_{40\%}$	1.6 ± 3.1	4.0 ± 5.3	-2.4 ± 2.7	-65.7 ± 14.7	0.001
	$D_{50\%}$	0.8 ± 1.6	2.2 ± 3.5	-1.3 ± 2.0	-58.9 ± 18.3	0.001

	D_{mean}	2.8 ± 2.7	5.6 ± 3.9	-2.8 ± 1.8	-54.1 ± 14.4	0.001
	ID	11.4 ± 10.0	23.1 ± 14.5	-11.6 ± 7.6		
Hippocampus CL	$D_{0.03cm^3}$	12.5 ± 13.9	18.7 ± 15.4	-6.2 ± 4.2	-50.3 ± 25.7	0.001
	$D_{2\%}$	11.7 ± 13.2	17.7 ± 15.0	-6.1 ± 4.6	-51.9 ± 26.5	0.002
	$D_{50\%}$	0.5 ± 0.8	1.4 ± 2.2	-0.9 ± 1.5	-50.3 ± 31.2	0.003
	D_{mean}	1.8 ± 2.2	3.7 ± 3.9	-1.9 ± 2.1	-57.9 ± 15.8	0.001
	ID	3.7 ± 4.4	7.3 ± 7.2	-3.7 ± 3.9		
Hippocampus IL	$D_{0.03cm^3}$	19.9 ± 13.6	27.9 ± 12.1	-8.0 ± 5.0	-35.2 ± 20.4	0.001
	$D_{2\%}$	18.9 ± 13.4	27.3 ± 12.2	-8.3 ± 5.2	-37.0 ± 20.3	0.001
	$D_{50\%}$	2.0 ± 3.4	5.1 ± 6.0	-3.2 ± 3.1	-62.5 ± 18.4	0.001
	D_{mean}	3.9 ± 3.5	7.7 ± 4.9	-3.8 ± 2.4	-53.1 ± 14.8	0.001
	ID	7.8 ± 6.2	15.7 ± 9.3	-8.0 ± 5.2		
Pituitary	$D_{0.03cm^3}$	39.2 ± 8.1	41.1 ± 6.5	-1.8 ± 2.2	-5.2 ± 7.6	0.001
	$D_{2\%}$	40.1 ± 7.2	42.0 ± 5.6	-2.0 ± 2.2	-5.2 ± 7.0	0.001
	D_{mean}	36.3 ± 11.4	37.9 ± 10.1	-1.7 ± 1.7	-6.1 ± 8.8	0.003
	ID	14.6 ± 7.3	15.3 ± 7.2	-0.7 ± 0.6		
Chiasma	$D_{0.03cm^3}$	36.5 ± 12.3	37.6 ± 11.0	-1.1 ± 1.4	-5.3 ± 9.0	0.007
	$D_{2\%}$	37.3 ± 10.5	37.8 ± 10.0	-0.6 ± 1.1	-2.3 ± 4.0	0.102
	D_{mean}	25.2 ± 15.0	27.0 ± 14.2	-1.7 ± 2.1	-11.5 ± 12.7	0.024
	ID	38.7 ± 26.6	41.8 ± 28.2	-3.1 ± 5.1		
Optic nerve CL	$D_{0.03cm^3}$	36.3 ± 10.8	36.9 ± 10.4	-0.6 ± 1.2	-2.2 ± 4.5	0.102
	$D_{2\%}$	36.7 ± 10.3	37.2 ± 9.9	-0.6 ± 1.1	-2.1 ± 3.8	0.123
	D_{mean}	26.2 ± 13.3	27.8 ± 12.3	-1.6 ± 2.0	-9.2 ± 12.2	0.010
	ID	30.6 ± 33.0	31.8 ± 32.1	-1.2 ± 1.9		
Optic nerve IL	$D_{0.03cm^3}$	41.8 ± 2.7	41.9 ± 2.9	-0.2 ± 0.6	-0.4 ± 1.5	0.377
	$D_{2\%}$	42.1 ± 2.4	42.3 ± 2.7	-0.3 ± 0.6	-0.6 ± 1.5	0.148
	D_{mean}	34.4 ± 9.0	35.4 ± 8.0	-1.0 ± 1.2	-3.6 ± 4.6	0.054
	ID	35.1 ± 28.0	35.8 ± 27.2	-0.7 ± 1.1		
Eye CL	$D_{0.03cm^3}$	14.2 ± 10.5	19.1 ± 9.0	-4.9 ± 4.1	-32.0 ± 29.8	0.001
	$D_{2\%}$	12.0 ± 9.5	16.4 ± 8.3	-4.4 ± 3.1	-34.5 ± 29.0	0.001
	D_{mean}	4.0 ± 3.6	6.1 ± 4.3	-2.1 ± 1.6	-43.0 ± 25.4	0.001
	$V_{10}(cm^3)$	1.0 ± 1.5	1.8 ± 2.2	-0.8 ± 0.9	-66.7 ± 31.4	0.001
	$V_{10}(\%)$	11.1 ± 15.6	19.1 ± 23.4	-8.0 ± 9.8		
	ID	36.9 ± 33.5	56.4 ± 41.3	-19.5 ± 15.6		
Eye IL	$D_{0.03cm^3}$	26.9 ± 12.4	28.6 ± 11.3	-1.6 ± 3.5	-8.3 ± 17.3	0.123
	$D_{2\%}$	24.2 ± 12.6	25.8 ± 11.4	-1.6 ± 3.1	-10.2 ± 17.9	0.102
	D_{mean}	9.4 ± 6.8	11.1 ± 7.2	-1.8 ± 1.3	-22.3 ± 17.9	0.001
	$V_{10}(cm^3)$	3.3 ± 2.7	4.0 ± 2.9	-0.7 ± 0.5	-35.5 ± 34.8	0.001
	$V_{10}(\%)$	36.6 ± 28.5	43.1 ± 30.2	-7.5 ± 5.9		
	ID	87.2 ± 65.0	103.4 ± 69.4	-16.2 ± 12.0		
Lens CL	$D_{0.03cm^3}$	2.3 ± 2.1	4.2 ± 3.6	-1.9 ± 2.2	-47.6 ± 25.1	0.001
	$D_{2\%}$	3.0 ± 3.0	5.1 ± 4.4	-2.1 ± 2.5	-45.4 ± 26.8	0.001
	D_{mean}	1.7 ± 1.8	3.2 ± 2.8	-1.5 ± 1.8	-47.9 ± 27.7	0.001
	ID	0.3 ± 0.3	0.7 ± 0.6	-0.3 ± 0.4		
Lens IL	$D_{0.03cm^3}$	4.9 ± 5.8	6.8 ± 7.3	-0.1 ± 1.9	-37.5 ± 20.2	0.001
	$D_{2\%}$	6.0 ± 6.9	8.0 ± 8.3	-2.1 ± 1.9	-34.5 ± 21.5	0.003

	D_{mean}	3.6 ± 4.2	5.3 ± 5.3	-1.6 ± 1.5	-39.8 ± 18.8	0.001
	ID	0.8 ± 1.0	1.1 ± 1.4	-0.4 ± 0.4		
Lacrimal gland CL	$D_{0.03cm^3}$	3.6 ± 4.0	5.5 ± 4.8	-1.9 ± 1.8	-41.6 ± 28.5	0.001
	$D_{2\%}$	3.8 ± 4.2	5.8 ± 4.7	-2.0 ± 1.9	-42.3 ± 28.7	0.005
	D_{mean}	1.8 ± 2.3	3.0 ± 3.6	-1.2 ± 1.7	-40.1 ± 37.5	0.007
	ID	1.9 ± 3.0	3.3 ± 5.5	-1.4 ± 2.6		
Lacrimal gland IL	$D_{0.03cm^3}$	10.1 ± 8.9	13.2 ± 10.4	-3.1 ± 2.7	-28.2 ± 18.3	0.001
	$D_{2\%}$	10.8 ± 9.3	14.0 ± 10.8	-3.1 ± 2.8	-26.9 ± 18.0	0.001
	D_{mean}	5.2 ± 4.9	7.1 ± 6.3	-1.9 ± 1.8	-31.3 ± 16.8	0.001
	ID	4.5 ± 5.3	6.3 ± 7.3	-1.8 ± 2.1		
Cochlea CL	$D_{0.03cm^3}$	4.9 ± 6.1	8.1 ± 6.7	-3.3 ± 2.6	-52.6 ± 24.1	0.002
	$D_{2\%}$	5.8 ± 6.7	9.5 ± 7.1	-3.7 ± 3.0	-49.3 ± 24.5	0.002
	D_{mean}	4.1 ± 5.4	7.3 ± 6.1	-3.1 ± 2.5	-55.3 ± 23.5	0.002
	ID	0.5 ± 0.7	0.9 ± 0.8	-0.4 ± 0.3		
Cochlea IL	$D_{0.03cm^3}$	17.1 ± 12.0	22.7 ± 13.7	-5.6 ± 4.5	-29.5 ± 17.2	0.001
	$D_{2\%}$	22.5 ± 13.8	26.5 ± 13.6	-4.0 ± 2.6	-20.4 ± 17.7	0.001
	D_{mean}	14.7 ± 10.3	20.7 ± 12.4	-5.9 ± 4.3	-32.9 ± 16.5	0.001
	ID	2.1 ± 2.2	2.8 ± 2.6	-0.8 ± 0.7		
Parotis CL	$D_{0.03cm^3}$	11.3 ± 5.5	16.0 ± 6.4	-4.7 ± 3.8	-29.5 ± 18.0	0.002
	$D_{2\%}$	9.5 ± 4.7	13.9 ± 5.8	-4.5 ± 4.0	-31.4 ± 20.8	0.005
	D_{mean}	3.9 ± 3.6	6.8 ± 4.1	-2.8 ± 2.2	-45.4 ± 23.6	0.002
	ID	74.7 ± 74.9	125.1 ± 93.7	-50.4 ± 45.3		
Parotis IL	$D_{0.03cm^3}$	23.1 ± 13.1	26.9 ± 11.9	-3.8 ± 2.8	-17.9 ± 13.3	0.001
	$D_{2\%}$	20.2 ± 13.4	23.2 ± 12.2	-3.0 ± 2.6	-17.3 ± 14.4	0.005
	D_{mean}	8.1 ± 7.9	10.6 ± 8.9	-2.5 ± 1.8	-31.4 ± 15.7	0.001
	ID	120.5 ± 109.8	159.4 ± 122.8	-38.9 ± 25.9		
Skin	$D_{0.03cm^3}$	32.2 ± 10.7	34.0 ± 9.3	-1.8 ± 2.4	-6.7 ± 9.0	0.019
	$D_{2\%}$	16.2 ± 6.6	20.3 ± 6.9	-4.1 ± 1.9	-21.2 ± 8.0	0.001
	$D_{5\%}$	10.4 ± 5.0	13.8 ± 5.2	-3.4 ± 0.8	-27.2 ± 8.5	0.001
	D_{mean}	1.6 ± 0.9	2.2 ± 1.1	-0.7 ± 0.2	-20.1 ± 27.2	0.001
	$V_{10}(cm^3)$	32.4 ± 27.9	49.4 ± 30.9	-17.0 ± 5.8	-31.2 ± 6.3	0.001
	$V_{15}(cm^3)$	15.3 ± 15.7	27.5 ± 26.5	-12.2 ± 11.9	-41.0 ± 15.4	0.001
	$V_{20}(cm^3)$	7.6 ± 8.5	13.2 ± 12.7	-5.6 ± 5.0	-51.7 ± 21.6	0.002
	$V_{35}(cm^3)$	1.1 ± 2.1	1.6 ± 3.1	-0.5 ± 0.9	-49.5 ± 25.2	0.063
	ID	876.4 ± 51.5.5	1235.5 ± 624.1	-359.1 ± 118.2		
