# Supplementary material for: Helium Ion Therapy for Advanced Juvenile Nasopharyngeal Angiofibroma
Source: Cancers (Basel). 2024 May 24;16(11):1993. doi: 10.3390/cancers16111993 (PMC11171253; doi:10.3390/cancers16111993)
Supplement: Supplementary file 1 [file cancers-16-01993-s001.zip › Supplementary Table S3.pdf]

**Supplementary Table S3**: Normal tissue complication probabilities (NTCP) for selected organs at risk, and risk ratio (RR) for secondary CNS malignancies. NTCP is expressed in %. IQ : intellectual quotient, GH : growth hormone, ACTH : adrenocorticotropic hormone, CL: contralateral, IL: ipsilateral, SD: standard deviation; Δabs: absolute difference.

| | | Helium | | | Proton | | | $\Delta_{abs}$(Helium - Proton) | | | |
|---|---|---|---|---|---|---|---|---|---|---|---|
| Organ at risk | Complication | Mean | ± | SD | Mean | ± | SD | Mean | ± | SD | p-value |
| Brain | IQ (Merchant et al. 2006) | 116.1 | ± | 4.6 | 115.6 | ± | 4.6 | 0.5 | ± | 0.3 | 0.001 |
| Brain | IQ (Mahajan et al. 2021) | 107.7 | ± | 0.2 | 107.6 | ± | 0.3 | 0.1 | ± | 0.1 | 0.001 |
| Brain | Neurocognitive impairment (IQ<85) | 1.5 | ± | 1.2 | 1.8 | ± | 1.3 | -0.3 | ± | 0.3 | 0.001 |
| Infratentorial Brain | IQ | 112.8 | ± | 3.9 | 112.4 | ± | 3.9 | 0.4 | ± | 0.2 | 0.001 |
| Supratentorial brain | IQ | 121.6 | ± | 5.6 | 121.2 | ± | 5.6 | 0.4 | ± | 0.2 | 0.001 |
| Cochlea CL | Tinnitus | 0.5 | ± | 0.5 | 0.7 | ± | 0.7 | -0.3 | ± | 0.3 | 0.002 |
| Cochlea CL | Hearing loss | 0.0 | ± | 0.0 | 0.0 | ± | 0.0 | 0.0 | ± | 0.0 | 1.000 |
| Cochlea IL | Tinnitus | 3.0 | ± | 3.6 | 7.0 | ± | 7.6 | -4.0 | ± | 5.3 | 0.001 |
| Cochlea IL | Hearing loss | 0.0 | ± | 0.0 | 0.0 | ± | 0.1 | 0.0 | ± | 0.1 | 0.063 |
| Hippocampus (bilateral) | Delayed recall | 4.6 | ± | 3.5 | 8.0 | ± | 10.2 | -3.5 | ± | 6.9 | 0.001 |
| Pituitary | Endocrine dysfunction | 1.0 | ± | 0.9 | 1.3 | ± | 1.1 | -0.3 | ± | 0.4 | 0.020 |
| Pituitary | GH-deficiency | 60.4 | ± | 20.5 | 63.1 | ± | 19.1 | -2.7 | ± | 2.2 | 0.003 |
| Pituitary | Hypothyroidism | 41.5 | ± | 18.1 | 44.1 | ± | 17.6 | -2.6 | ± | 1.9 | 0.003 |
| Pituitary | ACTH-deficiency | 23.7 | ± | 9.0 | 25.0 | ± | 8.8 | -1.4 | ± | 1.0 | 0.003 |
| Lacrimal gland IL | Ocular toxicity | 11.8 | ± | 18.5 | 18.1 | ± | 27.0 | -6.3 | ± | 8.6 | 0.001 |
| Lens CL | Cataract | 0.1 | ± | 0.2 | 0.7 | ± | 1.3 | -0.5 | ± | 1.3 | 0.001 |
| Lens IL | Cataract | 3.4 | ± | 10.5 | 7.4 | ± | 22.9 | -3.9 | ± | 12.9 | 0.001 |
| Parotis CL | Xerostomia | 1.4 | ± | 1.1 | 2.2 | ± | 1.6 | -0.8 | ± | 0.6 | 0.002 |
| Parotis IL | Xerostomia | 3.9 | ± | 5.5 | 5.6 | ± | 7.6 | -1.7 | ± | 2.7 | 0.001 |
| Skin | Alopecia | 38.0 | ± | 9.4 | 44.5 | ± | 10.2 | -6.4 | ± | 1.7 | 0.001 |
| Skin | Erythema | 18.7 | ± | 2.0 | 19.3 | ± | 3.1 | -0.6 | ± | 1.0 | 0.063 |

| | | RR (Proton/Helium) | | | |
|---|---|---|---|---|---|
| | | Mean | ± | SD | p-value |
| Brain | Secondary malignancies | 1.4 | ± | 0.2 | 0.001 |
